# Expression of DNA repair and replication genes in non-small cell lung cancer (NSCLC): a role for thymidylate synthetase (TYMS)

**DOI:** 10.1186/1471-2407-12-342

**Published:** 2012-08-06

**Authors:** Vassiliki Kotoula, Dimitrios Krikelis, Vasilios Karavasilis, Triantafillia Koletsa, Anastasia G Eleftheraki, Despina Televantou, Christos Christodoulou, Stefanos Dimoudis, Ippokratis Korantzis, Dimitrios Pectasides, Konstantinos N Syrigos, Paris A Kosmidis, George Fountzilas

**Affiliations:** 1 Department of Pathology, Aristotle University of Thessaloniki School of Medicine, Thessaloniki, Greece; 2 Laboratory of Molecular Oncology, Hellenic Foundation for Cancer Research, Aristotle University of Thessaloniki School of Medicine, Thessaloniki, Greece; 3 Department of Medical Oncology, “Papageorgiou” Hospital, Aristotle University of Thessaloniki School of Medicine, Thessaloniki, Greece; 4 Section of Biostatistics, Hellenic Cooperative Oncology Group, Data Office, Athens, Greece; 5 Second Department of Medical Oncology, “Metropolitan” Hospital, Piraeus, Greece; 6 Oncology Section, Second Department of Internal Medicine, “Hippokration” Hospital, Athens, Greece; 7 Oncology Unit GPP, "Sotiria" General Hospital, Athens School of Medicine, Athens, Greece; 8 Second Department of Medical Oncology, Hygeia Hospital, Athens, Greece

**Keywords:** NSCLC, ERCC1, TYMS, mRNA, Predictive, Normal lung, FFPE

## Abstract

**Background:**

BRCA1 (B), ERCC1 (E), RRM1 (R) and TYMS (T) mRNA expression has been extensively studied with respect to NSCLC patient outcome upon various chemotherapy agents. However, these markers have not been introduced into clinical practice yet. One of the reasons seems to be lack of a standard approach for the classification of the reported high/low mRNA expression. The aim of this study was to determine the prognostic/predictive impact of B, E, R, T in routinely-treated NSCLC patients by taking into account the expression of these genes in the normal lung parenchyma.

**Methods:**

B, E, R, T mRNA expression was examined in 276 NSCLC samples (real-time PCR). The normal range of B, E, R, T transcript levels was first determined in matched tumor – normal pairs and then applied to the entire tumor series. Four main chemotherapy categories were examined: taxanes-without-platinum (Tax); platinum-without-taxanes (Plat); taxanes/platinum doublets (Tax/Plat); and, all-other combinations.

**Results:**

In comparison to remotely located normal lung parenchyma, B, E, R, T mRNA expression was generally increased in matched tumors, as well as in the entire tumor series. Therefore, tumors were classified as expressing normal or aberrant B, E, R, T mRNA. In general, no marker was associated with overall and progression free survival (OS, PFS). Upon multivariate analysis, aberrant intratumoral TYMS predicted for shorter PFS than normal TYMS in 1st line chemo-naïve treated patients (p = 0.012). In the same setting, specific interactions were observed for aberrant TYMS with Plat and Tax/Plat (p = 0.003 and p = 0.006, respectively). Corresponding patients had longer PFS in comparison to those treated with Tax (Plat: HR = 0.234, 95% CI:0.108-0.506, Wald’s p < 0.0001; Tax/Plat: HR = 0.242, 95% CI:0.131-0.447, Wald’s p < 0.0001). Similar results were obtained for PFS in 1st line chemo-naïve and (neo)adjuvant pre-treated patients. Adenocarcinoma, early disease stage, and treatment with Tax/Plat doublets independently predicted for prolonged OS in patients who received only one line of treatment (adjuvant or 1st line).

**Conclusion:**

Classifying intratumoral B, E, R, T mRNA expression in comparison to normal lung may facilitate standardization of these parameters for prospective studies. With this approach, NSCLC patients with aberrant intratumoral TYMS expression will probably fare better with platinum-based treatments.

## Background

Current cytotoxic chemotherapy for NSCLC patients mostly includes doublets of platinum (alkylating agents causing DNA adducts) with taxanes or vinorelbine (plant alkaloids interfering with the stability of tubulins), with gemcitabine (a nucleoside analog interfering with DNA and RNA synthesis) or with pemetrexed (a folate antimetabolite). These are administered following surgical removal of the tumor (adjuvant setting), preceding surgical intervention (neoadjuvant) and in inoperable cases or metastatic disease (1^st^ line treatment). Cytotoxic chemo-therapeutics are applied based on in vitro and preclinical evidence of efficient cancer cell killing; after acceptable tolerance by the patients is ensured, drug combinations are mostly chosen empirically and cannot be distinguished for their efficacy in NSCLC patient outcome [1].

In an effort to rationalize treatment, predictive markers for cytotoxic drug efficacy in NSCLC patients have been extensively investigated over the last 15 years. Such markers mostly include molecules responsible for DNA synthesis and repair. With respect to DNA repair genes, low ERCC1 (excision repair cross-complementing rodent repair deficiency, complementation group −1) expression has been associated with a better outcome upon platinum compounds initially in patients with ovarian cancer [2] and later in patients with NSCLC [3-11]. High BRCA1 expression was associated with a better response to taxanes and sensitivity to docetaxel or docetaxel/gemcitabine (predictive value) [12,13] and with a worse overall survival in NSCLC patients both in early and advanced disease setting (prognostic value) [14,15]. With respect to DNA synthesis genes, low expression of ribonucleotide reductase M1 (RRM1), one of the two subunits of an enzyme essential for the production of deoxyribonucleotides prior to DNA synthesis in S phase of dividing cells, was associated with clinical benefit from neoadjuvant cisplatin/gemcitabine in NSCLC patients [7,11,16]. Thymidylate synthetase (TYMS), an enzyme that is critical in maintaining the dTMP (thymidine-5-prime monophosphate) pool for DNA synthesis and repair, is considered among the targets of newer antifolate drugs, such as pemetrexed [17,18], while allelic variants of TYMS have been shown to interfere with platinum activity in vitro [19]. In most studies, high TYMS expression is reported as an unfavourable prognostic factor [20-23], although it was occasionally associated with favourable outcome [24].

As yet, however, no conclusive evidence has been provided on the prognostic and/or predictive value of ERCC1, RRM1, BRCA1 and less of TYMS, nor has any consensus been reached on how these markers could be used for the routine assessment of NSCLC patients (critically reviewed in [25-28]). Reasons for this discrepancy include, among others, study design [29], method and methodology differences [25,27,30], type of tissue specimen [31], and primary or metastatic origin of the tissue examined [32,33]. Beyond methodological issues, the inability of comprehending the value of BRCA1, ERCC1, RRM1, and TYMS (B, E, R, T) expression in the assessment of NSCLC patients may be related to the nature of these markers. B, E, R, T are in fact expressed in the normal lung tissue in the frame of normal regeneration [34-36], and might thus contribute to the generic response of patients to cytotoxic therapy, which is given systemically and does not target tissues or tumors specifically.

In the present study, we investigated the prognostic/predictive impact of single and combined B, E, R, T mRNA expression profiles on the outcome of NSCLC patients who had been treated with platinum- and/or taxane-containing schemes in the adjuvant and 1^st^ line setting. Single and combined intratumoral B, E, R, T expression was compared with clinicopathologic parameters and interactions with treatment type in relevance to outcome. Other than has been practiced as yet though, we evaluated B, E, R, T expression in NSCLC in comparison to normal lung tissue located distally to the tumor site, in order to mitigate the effects of the field cancerization phenomenon [37].

## Patients, materials and methods

**Figure 1:**
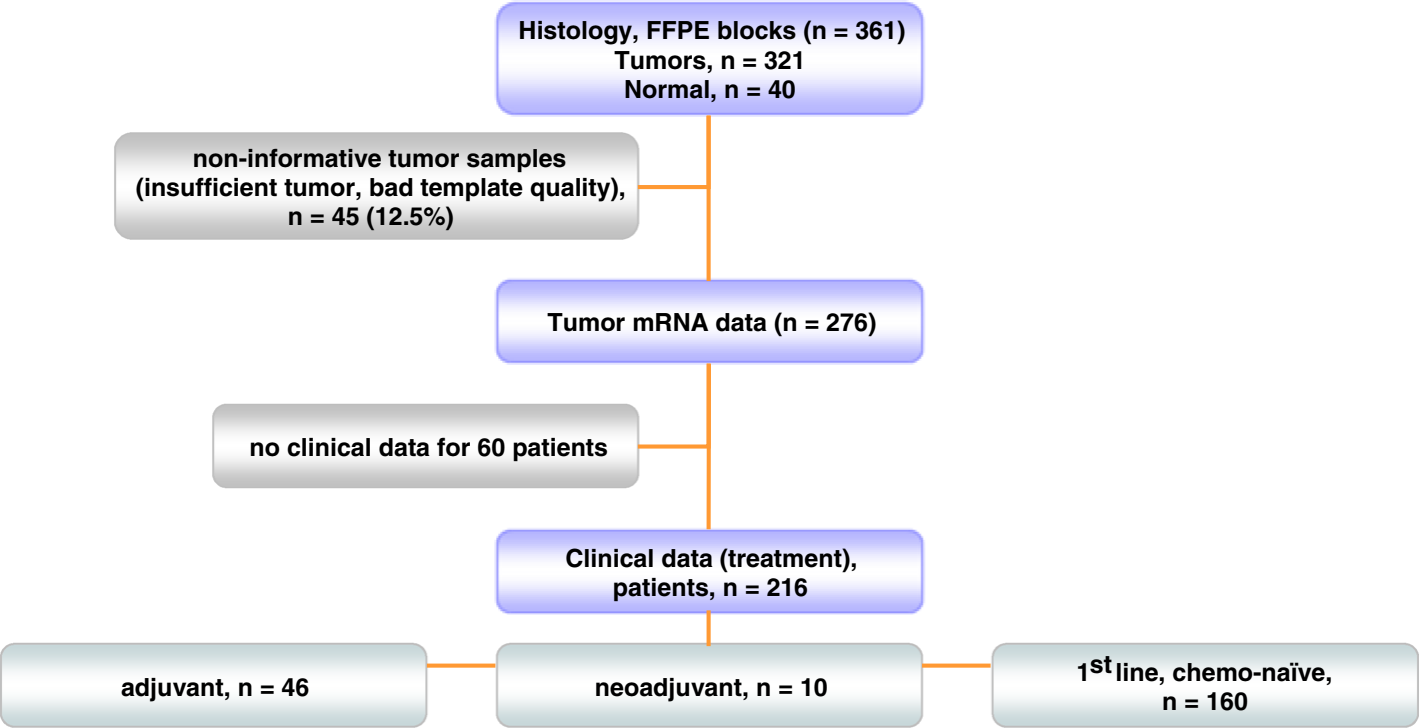
REMARK diagram of the tumors and patient populations studied.

The outline of tissue material and patient data involved in this study is presented in the REMARK diagram in Figure 1**.** In total, 361 NSCLC tumors were examined, for which histologic material (routinely diagnosed formalin-fixed paraffin-embedded tissue [FFPE] material) was available. Tissue specimens were collected from the Tumor Tissue Repository of Hellenic Cooperative Oncology Group (HeCOG). Corresponding demographic, clinicopathological and follow-up data had been registered for these patients in the frame of clinical service in HeCOG-affiliated hospitals. All patients had signed an informed consent form permitting the use of their biologic material for research purposes. The study was approved by the Bioethics Committee, School of Medicine, Aristotle University of Thessaloniki.

The clinicopathologic characteristics of registered patients and tumors are listed in Additional File 1 Table 1. Patients were considered as never, current and former smokers, the latter if they had quit the habit more than one year before treatment initiation. Throughout this manuscript, stage corresponds to disease stage at initial diagnosis. The treatment regimens administered and the corresponding patients’ outcome are shown in Additional File 1 Table 2. In order to examine homogeneous groups of patients we analyzed the following three subgroups; Subgroup A, including 1^st^ line treated chemo-naïve patients who initially presented with advanced disease (n = 160); Subgroup B, including all 1^st^ line treated patients (1^st^ line chemo-naïve and patients who relapsed upon adjuvant or neoadjuvant treatment) (n = 180); and Subgroup C, including chemo-naïve patients who were treated either in the (neo)adjuvant or metastatic setting (n = 192).

### FFPE tissue material

Involved previously diagnosed surgical specimens from lobectomy / pneumonectomy or from guided biopsies (total number of tumors: 326). Paraffin blocks with lung parenchyma without pathologic alterations (morphologically normal), distally located to the co-existing tumor, were available in 40 cases (lobectomy / pneumonectomy). Only primary tumors were included in this study, for the sake of molecular data uniformity, since the number of cases with available metastatic material was very small for comparisons (13 cases only). Three main histologic types were registered, adenocarcinomas (including bronchoalveolar carcinomas), squamous cell carcinomas (SCC) and large cell (LCC)/undifferentiated carcinomas. Based on a marked H&E section, macrodissection was performed where possible for tumor cell enrichment; tumor cell content was ≥50% in 308 tumors. Deparaffinized tissue fragments were digested overnight at 56^o^ C in a lysis buffer containing 10 mM NaCl, 50 mM Tris–HCl, pH 7.4, 20 mM EDTA, 1% SDS, and 0.8 mg/ml proteinase K. RNA was extracted from tissue lysates with TRIZOL-LS (Invitrogen / Life Technologies) and reverse transcribed with Superscript III and random hexamers (Invitrogen / Life Technologies), according to the manufacturers’ instructions. cDNAs were normalized at 25 ng/ul and stored at -20^o^ C until use.

mRNA expression was evaluated for BRCA1 (B), ERCC1 (E), RRM1 (R), and TYMS (T) with real time PCR. The relative expression of BRCA1 (Hs01556190_m1, amplicon included in all official BRCA1 splice variants); ERCC1 (Hs01012159_m1, all official ERCC1 splice variants); RRM1 (Hs00168784_m1); and TYMS (Hs00426591_m1) were assessed in comparison to GUSB (beta-glucuronidase, a housekeeping gene [#4333767 F]) with exon spanning, premade Taqman MGB expression assays (Applied Biosystems) in an ABI7500 real time PCR system under default conditions. GUSB was selected as the endogenous reference since, among the widely used housekeeping genes, it does not seem to be represented in pseudogenes. In addition, GUSB has been independently identified as one among the best preserved mRNA targets in FFPE tissues [38,39] with low variation in lung tissues [38]. A commercially available reference RNA derived from multiple transformed cell lines (TaqMan® Control Total RNA, cat. no 4307281, Applied Biosystems) was applied in multiple positions in each run as positive control and for inter-run evaluation of PCR assay efficiency. No-template controls were included. Samples were run in duplicates, at least in two metachronous runs. To obtain linear Relative Quantification (RQ) values, relative expression was assessed as (40-dCT), as previously described (Koutras, Kalogeras et al. 2008), whereby dCT (or deltaCT) was calculated as (average target CT) – (average GUSB CT) from all eligible measurements. Samples were considered eligible for analysis when both GUSB CTs in duplicates were <33 and when inter-run dCTs were <1. The reference RNA sample was used 34 times in parallel with tissue samples. The efficiency of all assays was considered as comparable, since the difference between inter-run RQ values for this sample was 0.64 for BRCA1; 0.33 for ERCC1; 0.89 for RRM1; and 0.69 for TYMS, marking for acceptable assay reproducibility. Based on the criteria described above for sample eligibility, informative results for B, E, R, T mRNA expression were obtained in 276/321 NSCLC tumor samples for ERCC1, RRM1 and TYMS, and in 274 for BRCA1 (approximately 86% of all tumor samples tested). Informative results were obtained in all 40 normal lung parenchyma samples but in 35 matched normal / tumor sample pairs (87.5%). Paired normal/tumor samples were always included in the same run.

### Statistics

Descriptive statistics include frequencies and corresponding percentages for binary variables and medians with range or means and standard deviations for continuous variables. Mann–Whitney or Kruskal-Wallis tests were used for comparisons between gene expression and clinicopathologic parameters; Spearman’s rank correlation for continuous RQ values in order to assess inter-gene expression associations. One sample *T*-test was used on the deltaRQ values (tumor RQ - normal RQ) in order to examine if gene expression was different in tumors and matched normal samples.

Progression-free survival (PFS) was measured from 1^st^ line treatment initiation until documented progression or death from any cause. Overall survival (OS) was measured from the date of initial diagnosis to the date of death or last follow-up. Time to event distributions were estimated by the Kaplan-Meier method and compared using the log-rank test. For the univariate and multivariate analyses, Cox proportional hazards models were used.

For Subgroup A we examined the prognostic significance of markers on PFS and OS, while for Subgroup B only for PFS and for Subgroup C only for OS. We also examined the predictive value of B, E, R, T mRNA expression by their interactions with the different chemotherapy regimens in the context of Cox regression models. In the multivariate setting, model choice was performed using backward selection criteria with removal criterion p > 0.10, including in the initial step basic clinicopathological parameters, such as: age, gender (women vs. men) smoking status (yes vs. no), tumor histology (other vs. adenocarcinoma) tumor stage (IIIb-IV vs. I-IIIa), and treatment regimen (platinum without taxanes vs. taxanes without platinum vs. platinum/taxanes combination vs. other). Possible interactions of markers with treatment regimen were included in the final model.

All tests were two-sided at the α = 0.05 level of significance. No adjustment for multiple comparisons was performed. The SPSS software was used for statistical analysis (SPSS for Windows, version 15.0, SPSS Inc.).

## Results

**Figure 2:**
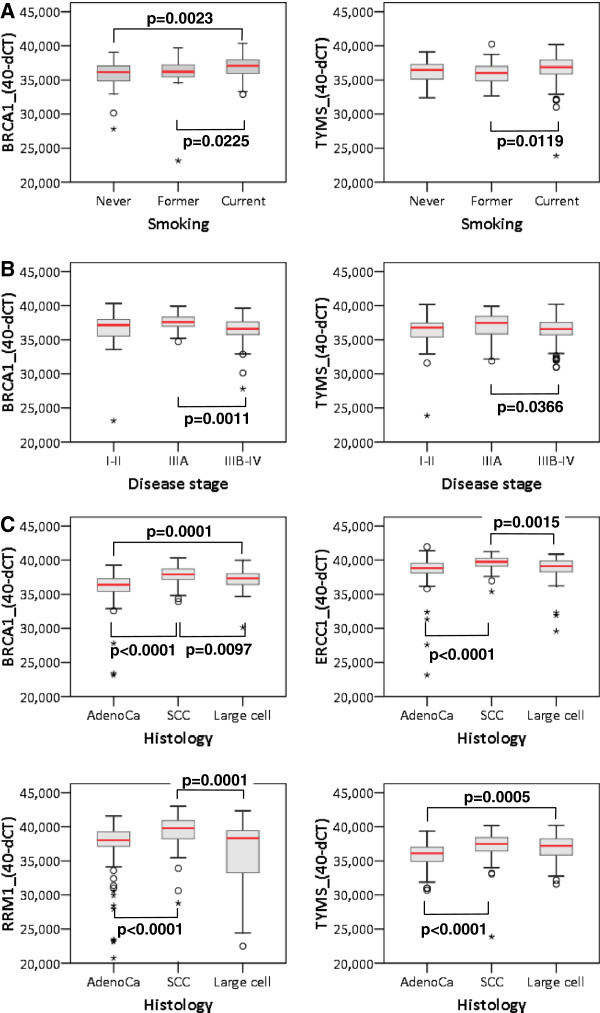
**Significant associations of clinicopathologic parameters with B, E, R, T mRNA expression.** Mann–Whitney significance is shown.

In tumors, B, E, R, T mRNA expression did not vary with age but it was higher in men than in women for BRCA1 and ERCC1 (Table 1). The expression of BRCA1 and TYMS was affected by smoking being higher in smokers than in never and former smokers (Figure 2A). Disease stage seemed to have the same impact on the expression of all genes, being higher in stage IIIA disease; however, significant differences were observed only for TYMS and, in particular, for BRCA1 (Figure 2B). Remarkably, B, E, R, T expression was significantly higher in SCC in comparison to adenocarcinomas and LCC, with adenocarcinomas expressing the lowest levels of these mRNAs (Figure 2C).

**Table 1:** Association of B, E, R, T mRNA expression with clinicopathologic parameters of NSCLC

		**BRCA1**		**ERCC1**		**RRM1**		**TYMS**	
	**N**	**median**	**range**	**median**	**range**	**median**	**range**	**median**	**range**
**All tumors#**		36.88	23.13−14.34	39.30	23.13−14.95	38.57	19.54−14.02	36.76	23.85−40.25
**Gender**									
Men	222	37.09	23.13−40.34	39.30	23.13−14.95	38.57	19.54−14.02	36.76	23.85−40.25
Women	54	36.24	32.95−39.22	38.22	35.81−40.87	37.95	30.60−41.94	36.51	30.99−39.88
*P*		*0.0053*		*0.0169*		*0.3615*		*0.4391*	
**Smoking**									
Never	29	36.16	27.80−39.06	39.01	27.58−40.45	38.19	19.54−41.23	36.45	32.33−39.07
Former	26	36.21	32.13−39.68	38.84	23.13−40.67	38.16	23.13−43.02	36.00	32.63−40.25
Current	150	37.08	32.34−40.34	39.36	32.27−41.95	38.84	23.34−43.02	36.00	32.63−40.25
*P*		*0.0017*		*0.2694*		*0.1541*		*0.0146*	
**Histology**									
Adeno	159	36.38	23.13−39.24	38.83	23.13−41.95	38.04	20.76−41.60	36.12	30.70−39.36
SCC	70	37.90	33.94−40.34	93.76	35.40−41.23	39.80	19.83−43.02	37.50	23.85−40.20
LCC/undiff	47	37.33	30.15−39.98	39.11	29.58−40.89	38.33	19.54−42.38	37.21	31.59−40.25
*P*		*<0.0001*		*<0.0001*		*<0.0001*		*<0.0001*	
**Stage**									
I & II	53	37.16	23.13−40.34	39.16	23.13−41.13	38.52	22.49−43.02	36.79	23.85−40.20
IIIA	34	37.60	34.74−39.96	39.70	36.22−41.23	39.30	30.60−42.26	37.48	31.89−40.20
IIIB & IV	137	36.61	27.80−39.68	39.26	27.58−41.95	38.54	19.54−42.38	36.57	30.99−40.25
*P*		*0.0064*		*0.5083*		*0.2249*		*0.1006*	

Notes to Table 1: Medians and range represent RQ values that are expressed as (40-dCT); p = Mann Whitney Asymptotic significance for gender and Kruskal-Wallis for the rest; # = the number of tumors with informative BRCA1 RQ values was 274, and for all other genes 276; undiff = undifferentiated; SCC = squamous cell, and LCC = large cell carcinoma; stage = stage at initial diagnosis.

The difference observed for BRCA1 and TYMS with respect to smoking status could not reliably be assessed in the normal lung samples, since 35 out of 40 of these corresponded to smokers. No difference in B, E, R, T mRNA expression was observed in this group with regard to age, gender and stage, while BRCA1 and TYMS expression was lower in the normal lung parenchyma of patients with adenocarcinomas in comparison to SCC (Mann–Whitney p = 0.001 in both cases).

Within tumor groups (entire series and tumors paired to normal samples), the same pattern of B, E, R, T mRNA expression was observed (Table 2), involving strong correlations of all markers to each other. In comparison, however, TYMS expression in normal lung samples was not related to BRCA1 or ERCC1, while it correlated positively with RRM1. These data indicate that, other than in the normal lung, common regulatory stimuli affect B, E, R, T mRNA expression in NSCLC tissues.

**Table 2:** Bivariate correlation of B, E, R, T mRNA expression in normal lung and NSCLC tissues

		**ERCC1**	**RRM1**	**TYMS**
**normal lung, n = 40**				
**BRCA1**	r	0.921	0.891	0.180
	*p*	*<0.001*	*<0.001*	*0.273*
**ERCC1**	r	1	0.933	0.244
	*p*		*<0.001*	*0.135*
**RRM1**	r		1	0.371
	*p*			*0.020*
**matched NSCLC,n = 35**				
**BRCA1**	r	0.919	0.858	0.560
	*p*	*<0.001*	*<0.001*	*<0.001*
**ERCC1**	r	1	0.904	0.564
	*p*		*<0.001*	*<0.001*
**RRM1**	r		1	0.628
	*p*			*<0.001*
**all NCLC,n = 276**				
**BRCA1**	r	0.522	0.383	0.511
	*p*	*<0.001*	*<0.001*	*<0.001*
**ERCC1**	r	1	0.625	0.350
	*p*		*<0.001*	*<0.001*
**RRM1**	r		1	0.358
	*p*			*<0.001*

Notes to Table 2: r = Spearman's Correlation Coefficient; *p* = Significance (2-tailed).

### B, E, R, T expression in NSCLC and matched normal lung parenchyma

**Figure 3:**
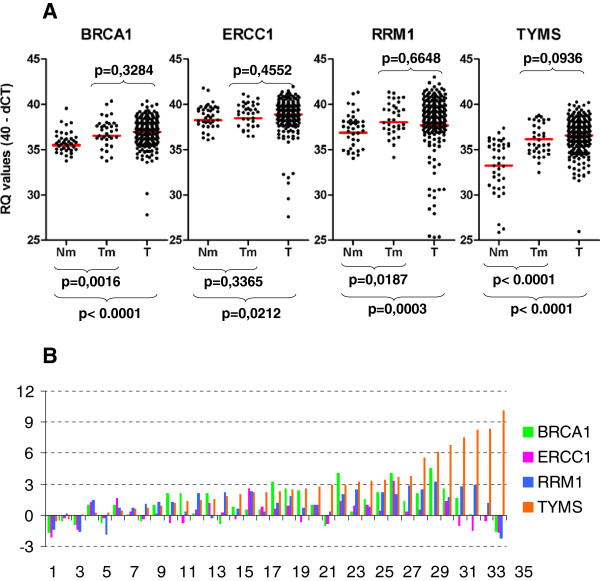
**BRCA1, ERCC1, RRM1 and TYMS (B, E, R, T) mRNA expression is up-regulated in NSCLC in comparison to non-cancerous lung tissue. A**. The distribution of single gene relative quantification (RQ) values is shown for non-cancerous lung tissue (Nm), matched tumor (Tm), and all remaining tumors for which no Nm was available (T). **B**. Comparison of B, E, R, T mRNA expression in a case-per-case mode. Except for very few cases, these genes, TYMS in particular, are up-regulated in NSCLC in comparison to matched non-cancerous lung tissue. A difference of 3 cycles (Y axis) corresponds to a 10-fold difference in gene expression.

A major problem with mRNA profiling for prospective applications is the definition of high versus low expression, since absolute quantification cannot be performed on FFPE tissue samples and measurements should always be compared to a standard. Herein, we first used the commercially available RNA reference as standard. As shown in Table 3, however, RRM1 and TYMS transcript levels were high in this sample. In comparison to this RNA reference, all tumors appeared as down-regulated for RRM1 and TYMS. Therefore, we assessed tumor B, E, R, T mRNA expression in reference to available paired tumor - normal lung samples. Tumors expressed significantly higher levels of BRCA1, RRM1 and TYMS transcripts in comparison to matched normal tissues, while ERCC1 mRNA expression was constant in normal and matched tumors (Table 3, Figure 3A and 3B). In line with these 35 matched pairs, B, E, R, T transcript levels were generally higher than normal in the entire group of NSCLC tissue samples examined (Table 3, Figure 3A).

**Table 3:** BRCA1, ERCC1, RRM1 and TYMS mRNA expression in the study sample groups

**sample category**	**BRCA1 mRNA**	**ERCC1 mRNA**	**RRM1 mRNA**	**TYMS mRNA**
**reference RNA, RQ**				
Mean	36.71	38.22	42.09	39.70
±SD	0.28	0.10	0.62	0.48
**normal lung tissue (N), RQ**	**40**	**40**	**40**	**40**
Mean	35.50	38.23	36.86	33.25
Median	35.66	38.63	37.47	34.10
±SD	2.46	2.93	3.02	2.94
Minimum	22.08	22.08	22.08	25.86
Maximum	39.54	41.78	41.32	37.37
**matched NSCLC tissue (T), RQ**	**35**	**35**	**35**	**35**
Mean	36.52	38.45	37.99	36.12
Median	36.98	39.08	38.27	35.57
±SD	2.79	2.95	3.13	1.70
Minimum	23.13	23.13	23.13	32.50
Maximum	40.34	41.13	41.34	38.77
**deltaTN^**	**35**	**35**	**35**	**35**
Mean	1.07	0.23	1.13	2.97
Median	1.00	0.31	1.20	2.38
±SD	1.60	1.19	1.37	2.07
Minimum	-1.66	-2.15	-2.24	-0.57
Maximum	4.55	3.38	3.23	10.17
* 95% C.I.*	*0.52 - 1.62*	*-0.18 - 0.64*	*0.66 - 1.60*	*-0.03 - 3.92*
* Sig., 2-tailed (one-sample T-test)*	*0.0004*	*0.2610*	*<0.0001*	*<0.0001*
**NSCLC tissue, non-matched, RQ**	**239**	**241**	**241**	**241**
Mean	36.75	38.87	37.59	36.44
Median	37.00	39.12	38.52	36.71
±SD	1.82	1.74	3.99	2.01
Minimum	23.40	27.58	19.54	23.85
Maximum	39.96	41.95	43.02	40.25

Notes to Table 3: ^: relative mRNA expression in matched Tumor (T) vs. Normal (N) tissues followed normal distribution.

**Figure 4:**
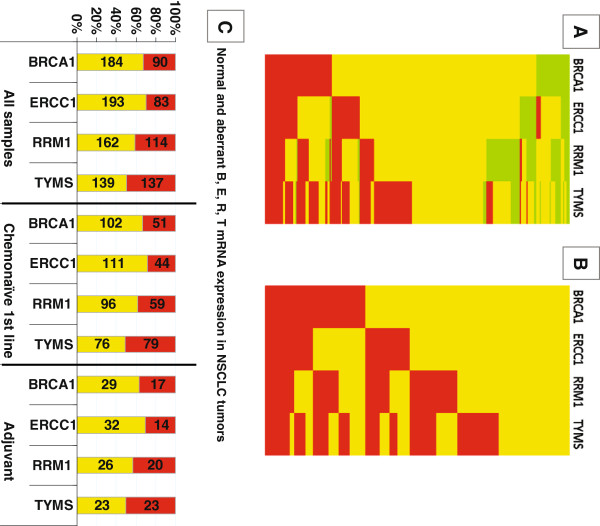
**B, E, R, T mRNA expression in NSCLC tumors.** In (**A**), distribution of normal (yellow), low (green) and high (red) mRNA expression is shown. The majority of tumors expressed normal RQ values for BRCA1, ERCC1 and RRM1, while in relatively few tumors the expression of all genes was low. (**B**): To be able to further analyze our cohort, we created a binary scale for RQ values, including all those aberrant from normal (yellow) in one category (red). (**C**): Classification of RQ values as normal and aberrant-from-normal yielded a comparable incidence of each category in the entire tumor series and in treatment related groups. Incidence (numbers within bars) and rates (Y-axis) of tumors with normal (yellow) and aberrant (red) relative expression values for each gene in three study groups are shown.

In order to set the normal range of B, E, R, T RQ values for scaling the corresponding mRNA expression in tumors, we used the highest populated 3-cycle range in each RQ series of normal samples (Additional 1 Table 3). The 3-cycle range approximately corresponded to the standard deviation observed for all normal measurements (Table 3). The normal RQ values for each gene thus ranged between 35–38 for BRCA1, 37–40 for ERCC1 and RRM1, and 34 – 37 for TYMS. The majority of tumors expressed normal BRCA1 (67.15%), ERCC1 (69.93%), RRM1 (58.70%) and TYMS (50.38%) transcript levels (Additional file 1 Table 4, Figure 4A). Scaling of tumor RQ values into lower than normal, normal, and higher than normal yielded low B, E, R, T expression in only 8.39%, 9.06%, 22.10% and 9.42% of the NSCLC tumors examined. This classification would hamper statistical analysis, since, for example, tumors with low B, E, R, T expression were not always included in adequate numbers in the treatment groups examined. Thus, we preferred to use the terms “normal” and “other than normal” (i.e., aberrant) for tumor RQ values. Aberrant RQ values for each B, E, R, T mRNA included both lower and higher expressing tumors. The distribution and profiles of this binary classification of B, E, R, T mRNA expression for all tumors is profiled in Figure 4B. The distribution of normal and aberrant RQ values in NSCLC samples was comparable in the major patient groups in this study (Figure 4C).

### Effect of B, E, R, T mRNA expression on NSCLC patient outcome

For the analysis of the impact of B, E, R, T RQ values on patient outcome, we initially used a 3-scale classification, i.e., lower than normal (low), normal, and higher than normal (high), whereby the normal category was defined as above. These results are shown in Additional file 1 Figure 1. It appeared that with respect to treatment, the markers had the same effect on patient outcome when aberrantly expressed, either low or high. These patterns were similar to, or different as compared to tumors with mRNA expression in the normal range for each marker. The latter condition was particularly pronounced for TYMS. These observations justified the merging of aberrant RQ values (low-high) for the same marker into one category.

Among all B, E, R, T binary mRNA parameters tested, none was associated with the overall survival of patients either in Subgroup A or in Subgroup C. Similarly, none of the markers was associated with PFS in Subgroups A and B of 1^st^ line-treated patients except from normal RRM1 mRNA levels-expressing tumors, which were associated with longer PFS in comparison to those with aberrant RRM1. However, this association did not reach statistical significance (Table 4).

**Table 4:** Univariate Cox regression analysis for B, E, R, T RQ values according to progression free and overall survival

	**aberrant vs. normal**	**Progression free survival**					**Overall survival**				
		**Total N**	**Events**	**HR**	**95% C.I.**	**Wald’s p**	**Total N**	**Events**	**HR**	**95% C.I.**	**Wald’s p**
**Subgroup A**	BRCA1	51 vs. 100	44 vs. 79	1.307	0.901-1.897	0.158	51 vs. 102	35 vs. 62	1.019	0.672-1.545	0.930
	ERCC1	43 vs. 110	36 vs. 88	1.224	0.829-1.806	0.309	44 vs. 111	30 vs. 68	1.169	0.760-1.799	0.478
	RRM1	58 vs. 95	49 vs. 75	1.348	0.936-1.939	0.108	59 vs. 96	42 vs. 56	1.141	0.760-1.715	0.524
	TYMS	78 vs. 75	63 vs. 61	0.849	0.593-1.216	0.373	79 vs. 76	50 vs. 48	0.871	0.583-1.302	0.500
**Subgroup B**	BRCA1	58 vs. 113	49 vs. 87	1.313	0.921-1.872	0.132					
	ERCC1	50 vs. 123	41 vs. 96	1.251	0.867-1.805	0.232					
	RRM1	66 vs. 107	55 vs. 82	1.371	0.971-1.935	0.073					
	TYMS	87 vs. 86	68 vs. 69	0.922	0.656-1.297	0.642					
**Subgroup C**	BRCA1						62 vs. 123	38 vs. 67	1.003	0.672-1.499	0.987
	ERCC1						53 vs. 134	32 vs. 74	1.02	0.672-1.548	0.926
	RRM1						73 vs. 114	45 vs. 61	1.059	0.718-1.562	0.772
	TYMS						95 vs. 92	56 vs. 50	1.027	0.699-1.509	0.892

Subgroup A: 1st line, chemo-naïve; Subgroup B: 1st line, chemo-naïve and pre-treated; Subgroup C: chemo-naïve, 1st line and non-relapsed adjuvant; empty cells: not-applicable.

**Figure 5:**
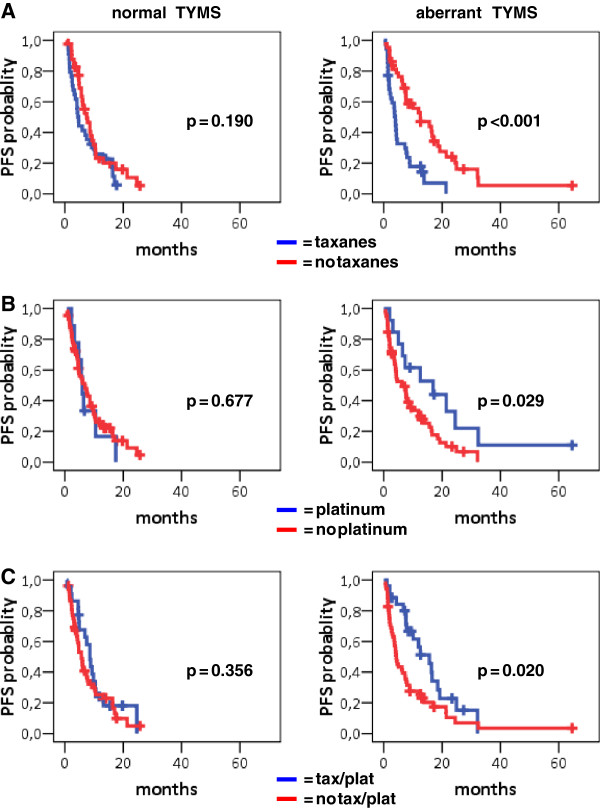
**TYMS mRNA expression is inversely related to the outcome of chemo-naïve 1st line treated patients who received taxanes and/or platinum containing schemes.** Normal TYMS was not predictive of PFS upon any of these treatments. In comparison, aberrant TYMS was associated with favorable PFS in patients who did not receive taxanes (**A**) and in particular, in patients who received platinum containing chemotherapy without taxanes (**B**) or in combination with taxanes (**C**). In patients who received taxanes in any other combination except for platinum, aberrant TYMS was associated with poor PFS, as shown in A.

Interactions of B, E, R, T mRNA expression with the effect of drugs administered could not be assessed in the adjuvant setting, where out of 46 patients, 41 had received taxanes and 44 platinum-containing regimens. We also examined possible interactions of B, E, R, T markers with the drugs administered in the 1^st^ line or adjuvant setting. A significant interaction of TYMS with taxanes was found in both 1^st^ line cohorts (Subgroup A p = 0.038; Subgroup B p = 0.022), while the interaction of TYMS with platinum was significant in Subgroup B only (p = 0.030). More specifically, in 1^st^ line chemo naïve patients (Subgroup A), TYMS was significantly associated with patient outcome in relation to specific drugs or regimens. When excluding the taxanes/platinum combination, patients with intratumoral aberrant TYMS performed better when they did not receive taxanes, in comparison to those who received taxanes (Table 5, Figure 5A). Similarly, intratumoral aberrant TYMS predicted for a favorable PFS in patients who received platinum without taxanes in comparison to those who did not receive platinum (Figure 5B). In patients who did receive the taxanes/platinum combination, aberrant intratumoral TYMS was again associated with prolonged PFS in comparison to those who did not receive the combination (Figure 5C). Identical results were obtained with respect to aberrant TYMS in Subgroup B.

**Table 5:** TYMS mRNA expression was associated with patient progression free survival

		**Progression free survival (months)**					
		**Total N**	**Events**	**Median**	**HR**	**95% C.I.**	**Wald’s p**
**Subgroup A**	**Aberrant TYMS**						
	No taxanes vs. Taxanes	43 vs. 35	32 vs. 31	12.53 vs. 4.00	0.335	0.199-0.565	<0.001
	No platinum vs. platinum	66 vs. 13	53 vs. 10	6.72 vs. 16.92	2.247	1.097-4.602	0.027
	No tax/plat vs. tax/plat	52 vs. 26	45 vs. 18	4.26 vs 15.64	1.982	1.143-3.437	0.015
	**Normal TYMS**						
	No taxanes vs. Taxanes	41 vs. 34	32 vs. 29	7.48 vs. 4.39	0.716	0.431-1.190	0.197
	No platinum vs. platinum	66 vs. 9	53 vs. 8	6.89 vs. 5.97	0.844	0.400-1.779	0.656
	No tax/plat vs. tax/plat	53 vs. 22	43 vs. 18	5.64 vs. 8.56	1.270	0.731-2.205	0.396
**Subgroup B**	**Aberrant TYMS**						
	No taxanes vs. Taxanes	51 vs. 36	36 vs. 32	12.53 vs. 3.77	0.349	0.212-0.575	<0.001
	No platinum vs. platinum	73 vs. 14	58 vs. 10	6.72 vs. 16.92	2.216	1.088-4.512	0.028
	No tax/plat vs. tax/plat	57 vs. 30	48 vs. 20	13.21 vs. 19.31	2.112	1.248-3.573	0.005
	**Normal TYMS**						
	No taxanes vs. Taxanes	47 vs. 39	36 vs. 33	6.89 vs. 5.93	0.771	0.479-1.242	0.286
	No platinum vs. platinum	76 vs. 10	60 vs. 9	7.44 vs. 5.64	0.731	0.362-1.478	0.383
	No tax/plat vs. tax/plat	62 vs. 24	49 vs. 20	12.18 vs. 12.85	1.049	0.623-1.767	0.857

BRCA1 and RRM1 mRNA expression appeared to be significantly associated with patient outcome following particular drug treatments in Subgroups A and B (Additional file 1 Figure 2). However, these associations were not specific and did not further yield drug-specific interactions with respect to outcome, which was the query of this study. On the contrary, ERCC1 mRNA expression was related to patients’ response according to treatment. In Subgroup A, as shown in Additional file 1 Figure 3, normal intratumoral ERCC1 RQ values were associated with a favorable PFS in those who did not receive taxanes excluding the taxanes/platinum combination (median 8.79 mo), in comparison to those who did (median 4.13 mo, Log-rank p < 0.001). Patients with tumors expressing aberrant ERCC1 having received platinum without taxanes also performed better (PFS, median 32.36 mo) in comparison to patients who received all other combinations (PFS, median 4.85 mo, Log-rank p = 0.039). However, the number of platinum-treated patients in this case was very small for reliable comparisons (n = 5). Finally, patients with normal ERCC1-expressing tumors who received taxanes/platinum combinations also had a longer PFS (median 10.36 mo) compared to those who were not treated with this regimen (median 4.75 mo, Log-rank p = 0.003). The latter finding was the only association that yielded a trend of drug-specific interaction for ERCC1 (Wald’s p = 0.087); patients with tumors expressing normal ERCC1 had favorable PFS when they were treated with taxanes/platinum combination (HR = 0.512, 95% CI 0.322-0.818, p = 0.05) compared to patients who did not receive the combination. For tumors with aberrant ERCC1 no difference was observed in the same treatment context. Although the associations observed individually for normal and aberrant ERCC1 were retained in Subgroup B, the interaction described above was not. We next tried to create ERCC1 and TYMS profiles but the number of patients treated with the schemes examined in each category was not adequate for reliable statistical analysis.

In multivariate analysis for PFS, the predictive role of TYMS interaction with taxanes remained significant in the final model for Subgroups A and B (Table 6). More specifically, patients with aberrant intratumoral TYMS who were treated with platinum or taxanes/platinum combination had a decreased risk for relapse compared to patients treated with taxanes without platinum (Subgroup A: HR = 0.234, 95% CI:0.108-0.506, Wald’s p < 0.0001 and HR = 0.242, 95% CI:0.131-0.447, Wald’s p < 0.0001, respectively; Subgroup B: HR = 0.256, 95% CI:0.141-0.466, Wald’s p < 0.0001 and HR = 0.240, 95% CI:0.111-0.521, Wald’s p < 0.0001, respectively). Concerning normal intratumoral TYMS no difference was observed for the above comparisons of treatment regimens. In terms of OS, TYMS was not predictive for treatment while women, adenocarcinoma histology, early disease stage (for Subgroup C only) and taxanes/platinum combination were independently associated with prolonged survival (Table 7).

**Table 6:** Multivariate analysis for progression free survival (PFS) in Subgroup A and Subgroup B

	**PFS**				
	**Parameter**	**SE**	**HR**	**95%CI**	**WAld’s p**
**Subgroup A(1st line, chemo-naïve)**					
Age	−0.028	0.011	0.973	0.952-0.994	0.012
TYMS					
aberrant vs. normal	0.577	0.253	1.780	1.084-2.923	0.563
Treatment					
platinum vs. taxanes	0.235	0.407	1.265	0.570-2.811	0.563
tax/plat vs. taxanes	−0.220	0.303	0.802	0.443-1.452	0.467
other vs. taxanes	0.088	0.376	1.092	0.522-2.282	0.816
aberrant TYMS *platinum	−1,687	0.564			0.003
aberrant TYMS *tax/plat	−1.200	0.433			0.006
**Subgroup B(1st line, chemo-naïve)**					
Age	−0.002	0.010	0.978	0.959-0.998	0.034
TYMS					
aberrant vs. normal	0.539	0.255	1.714	1.040-2.824	0.034
Treatment					
platinum vs. taxanes	0.293	0.382	1.340	0.0633-2.836	0.444
tax/plat vs. taxanes	−0.129	0.285	0.879	0.502-1.537	0.650
other vs. taxanes	−6.631	0.439	0.532	0.225-1.258	0.151
aberrant TYMS*platinum	−1.719	0.551			0.002
aberrant TYMS*tax/plat	−1.233	0.420			0.003
aberrant TYMS *other treatment	−1.375	0.642			0.032

**Table 7:** Multivariate analysis for overall survival (OS) in Subgroup A and Subgroup C

	**OS**				
	**Parameter**	**SE**	**HR**	**95% CI**	**Wald’s p**
**Subgroup A(1st line, chemo-naïve)**					
Gender					
Women vs. Men	−0.586	0.315	0.557	0.300-1.033	0.063
Histology					
Other vs. adenoCa	0.378	0.201	1.459	0.984-2.164	0.060
Treatment					
Platinum vs. taxanes	−0.306	0.272	0.736	0.432-1.255	0.260
tax/plat vs. taxanes	−1.098	0.233	0.334	0.211-0.527	<0.0001
Other vs. taxanes	−0.086	0.417	0.918	0.405-2.079	0.837
**Subgroup C (Chemo-naïve, 1st line and non-relapsed adjuvant)**					
Gender					
Women vs. Men	−0.552	0.311	0.576	0.313-1.058	0.075
Stage					
IIIb-IV vs. I-IIIa	1.224	0.267	3.399	1.136-2.576	<0.0001
Histology					
Other vs. adenoCa	0.549	0.203	1.731	1.136-2.576	0.007
Treatment					
Platinum vs. taxanes	−0.174	0.271	0.840	0.494-1.430	0.522
tax/plat vs. taxanes	−0.647	0.247	0.524	0.323-0.849	0.009
other vs. taxanes	0.042	0.415	1.043	0.463-2.350	0.920

## Discussion

So far, successful drug-specific predictive markers in NSCLC and in most malignancies include molecular phenotypes reflecting somatic genetic alterations; for example, small scale mutations or large scale structural gene changes. Regarding BRCA1, ERCC1, RRM1 and TYMS in NSCLC, however, differences in the *“expression level”* of the respective molecules are considered as markers, usually by referring to high vs. low expression. High vs. low is a qualitative description deriving from (semi)quantitative measurements in the case of q-PCR gene expression assessments. Undoubtedly, in order to understand high vs. low we need to refer to a standard. By using an external “normal” cell line RNA as a standard (as published before for ERCC1) [3], two of these genes, RRM1 and TYMS, appeared as very low expressed in tumors. However, even when coined as “normal”, cells in culture are, in fact, transformed; therefore, having acquired the ability of continuous division they inherently produce enzymes such as RRM1 and TYMS. In addition, cells in culture constitute a homogeneous system by definition. In comparison, cells in normal tissues comprise a heterogeneous environment and divide upon interaction with their surrounding cells. This explains the very low expression of RRM1 and TYMS in the normal lung tissue in comparison to the reference standard, as observed here.

Our approach was to use normal lung tissues located distally to tumors as a standard. Given the retrospective type of this study, it was difficult to find a large number of such normal samples with matched tumors. The limited number of samples in this reference group (12.5% of the total number of cases examined) may be considered as a drawback for the normal range of RQ values obtained in this study. However, with respect to the RQ values in the matched tumor samples at least, the group was considered representative for the remaining 87.5% of single tumor samples. The level of BRCA1, ERCC1, RRM1 and TYMS transcripts in NSCLC tumors of all histologic types, and especially SCC, was mostly within the normal range or increased. These observations are in line with previous reports for unchanged ERCC1 [36] or increased ERCC1 [35] and for increased TYMS mRNA expression [40,41] in tumors vs. normal lung, whereby TYMS has also been associated with enhanced proliferation activity [22]. Genetic or epigenetic BRCA1 silencing in NSCLC could not be inferred from our study, contrary to a previous report [34], since only two tumors in our series exhibited very low expression of this gene. RRM1 mRNA expression was found lower in NSCLC compared to the normal lung and the condition was attributed, at least in part, to LOH at 11p15.5 [16], which may also have accounted for the low RRM1 observed in 22% of tumors in this study. For BRCA1, ERCC1 and TYMS, expression below the normal range was encountered in <10% of the tumors examined. Overall, only in 11 cases (<4%) tumor B, E, R, T RQ values were lower than any normal ones, which would indicate absence of gene expression. Thus, although genetic / epigenetic changes may have been present in these cases, low expression of B, E, R, T indicative of gene pathology in NSCLC tumors could not be considered as a frequent event. In addition, considering that all mRNA markers strongly correlated with each other in tumors, our findings on mRNA expression of the two DNA-repair and the two DNA-replication genes in NSCLC reflect the expected patterns of tissues responding to increased demands for both these cell functions. Of note, our results are limited to gene expression, which does not preclude that for example, these particular DNA repair genes would be dysfunctional in NSCLC. BRCA1 and ERCC1 mRNA expression has as yet been addressed in a rather simplistic way in tissue studies with q-PCR, abolishing the possibility to investigate which mRNA variants are being produced from these genes. At least for ERCC1, detection of a particular splice variant might be important for the evaluation of platinum resistance, as has been reported for ovarian cancer cells [42].

In the noticeably heterogeneous patient cohort of the present study, none of the four markers was universally associated with OS and DFS in the adjuvant setting or PFS in the two different first-line settings. This finding was rather expected, since the prognostic value of the markers examined remains contradictory, as supported in recent meta-analyses for ERCC1, which also stress the need for standardization methods to assess gene expression by qPCR in tumors [27,30]. The only independent predictors for OS, after adjusting the four mRNA markers for clinicopathologic parameters and treatment schemes, were disease stage (unfavorable if advanced) and histology type, meaning adenocarcinomas, which conferred a favorable outcome in comparison to squamous and large cell carcinomas. However, this latter finding must be considered as cohort specific, since the majority of patients in this study were chemo-naïve adenocarcinoma patients and received 1^st^ line chemotherapy, where adenocarcinomas are the best responders among NSCLC [43]. No interaction between histology type and BRCA1, ERCC1, RRM1, or TYMS transcript levels was observed in terms of outcome, despite the strong association of continuous RQ values with the same parameter. The relative expression of the four genes was similar in adeno- and large cell carcinomas but, in comparison to these, it was significantly increased in squamous cell carcinomas, in line with previously published data [4,40]. Apparently, the expression of these genes did not predict for the outcome of adenocarcinoma patients in our series, in line with recent reports with immunohistochemistry for Ercc1 and Brca1 [44,45].

The heterogeneous treatment regimens administered for advanced disease in this study constituted a very useful template to assess drug-specific interactions of BRCA1, ERCC1, RRM1, and TYMS, particularly with respect to patients’ PFS, an endpoint which is closely related to treatment efficacy although more prone to errors during assessment [46]. It was interesting to observe the same treatment-related patterns of patient outcome for tumors with low and high expression of the markers examined, while tumors with normal expression of the same marker had different prognosis. Plausibly, the first approach for the interpretation of this finding would be to consider it an artifact due to the small number of tumors with low BRCA1, ERCC1, RRM1, and TYMS mRNA expression. However, this finding may also suggest that drug interactions, if any, with the molecules examined at the protein level may follow bell-shaped curves, as known for various biologic response models involving enzymes (e.g., concerning the cellular fate upon different levels of superoxide dismutase [47]) or drug efficiency [48]. To prove this, functional studies in controlled systems with multicellular components are needed. It remains worthy considering, however, that the usually applied one-point cut-offs may mask the information needed for the evaluation of the herein investigated as well as for other mRNA markers.

In this context, normal ERCC1 transcript levels were associated with a favorable outcome in patients who received the platinum/taxanes doublet. Considering that normal ERCC1 mostly corresponds to low ERCC1 as addressed in the literature, this finding was in keeping with the reported platinum specificity of low ERCC1 [27,30,49]. However, a clear effect as would be expected for normal ERCC1 on patients treated with platinum-based regimens without taxanes was not observed in the present study.

Surprisingly, TYMS followed the same predictive pattern of significance as ERCC1 for platinum without taxanes, but an inverse pattern than ERCC1 for taxanes without platinum. For this molecule, significant results were obtained with respect to platinum (high TYMS – favorable) and taxanes (high TYMS – unfavorable), and less so for the combination of the two drugs (high TYMS – favorable). Since the sole independently significant interaction for patient outcome was TYMS with taxanes, patients with tumors expressing high TYMS mRNA, as compared to a normal lung tissue standard, may be spared receiving taxanes. This is new evidence, meriting clarification at the molecular level for the known microtubule stabilizers. In addition, there was an interaction of high TYMS with platinum as well, probably reflecting the increased efficacy of this drug in killing cells featuring active DNA replication. TYMS, an enzyme that is essential for DNA synthesis, has not yet been studied as a “platinum-target” molecule, as has been ERCC1 in the context of the nucleotide excision repair pathway. In NSCLC therapeutics, TYMS counts as an established target mostly being associated with the efficacy of the newer antifolate pemetrexed, which is a multi-targeted drug, nonetheless [50,51]. Another observation from the present study concerns the widely used taxanes/platinum combination in advanced NSCLC patients. ERCC1 and TYMS were related in an opposing manner to patient outcome upon this treatment, with TYMS being more specific. It would be interesting to investigate the drug-specific predictive value of combined profiles of ERCC1 and TYMS in a larger series of patients with adequate statistical power.

## Conclusions

This study shows that the expression of genes participating in DNA repair and replication are mostly up-regulated in NSCLC, as would be expected for proliferating cells, without indications for a severe dysfunction of these two cellular functions at the mRNA level. To further elucidate the cellular molecular involvement in cytotoxic drug targeting, an extended cohort of NSCLC patients is being treated with the Docetaxel / Carboplatin combination (plus bevacizumab when indicated) in the 1^st^-line setting in HeCOG-affiliated hospitals. Detailed biomarker studies and correlations with outcome in this cohort are expected to shed more light on the interaction of DNA replication and repair molecules with chemotherapy in the quest of refining current treatment strategies for patients with NSCLC.

## Competing interests

The authors declare no competing interests.

## Authors' contributions

VK: study design, molecular investigations, data analysis and interpretation, manuscript writing; DK: data analysis and interpretation, manuscript writing; VK: provision of clinical data, data interpretation, critical review of manuscript; TK: histologic evaluation of tissue material, manuscript review; AGE: statistical analysis, manuscript writing; DT: histologic evaluation of tissue material, manuscript review; CC: provision of clinical data, manuscript review; SD: provision of clinical data, manuscript review; IK: data interpretation, manuscript review; DP: provision of clinical data, manuscript review; KNS: provision of clinical data, manuscript review; PAK: provision of clinical data, manuscript review; GF: study conception and design, provision and supervision of clinical data and tissue samples, data interpretation, critical review of manuscript. All authors have read and approved the final version of the manuscript.

## Pre-publication history

The pre-publication history for this paper can be accessed here:

http://www.biomedcentral.com/1471-2407/12/342/prepub

## Supplementary Material

Additional file 1 **Table 1:Clinicopathologic characteristics of NSCLC patients and tumors examined.Table 2:** Treatment-related patient groups and outcome. **Table 3:** Selection of the normal RQ value range was based on the highest frequency of RQ values within a 3-cycle range in the normal sample series. **Table 4:** Initial classification of B, E, R, T RQ values in NSCLC tumors. **Figure 1:** PFS patterns of patients in Subgroup B (1^st^ line, chemo-naïve + pre-treated) with mRNA RQ values classified in a 3-scale as low, normal and high. **Figure 2:** Significant but non-specific associations of BRCA1 and RRM1 gene expression with the outcome of 1^st^ line chemo-naïve patients treated with taxanes excluding the platinum doublet. **Figure 3:** Effect of ERCC1 mRNA expression on the outcome of 1^st^ line chemo-naïve treated patients. Click here for file
