# Supplementary material for: Expression of DNA repair and replication genes in non-small cell lung cancer (NSCLC): a role for thymidylate synthetase (TYMS)
Source: BMC Cancer. 2012 Aug 6;12:342. doi: 10.1186/1471-2407-12-342 (PMC3503623; doi:10.1186/1471-2407-12-342)
Supplement: Additional file 1 — Table 1:Clinicopathologic characteristics of NSCLC patients and tumors examined.Table 2: Treatment-related patient groups and outcome. Table 3: Selection of the normal RQ value range was based on the highest frequency of RQ values within a 3-cycle range in the normal sample series. Table 4: Initial classification of B, E, R, T RQ values in NSCLC tumors. Figure 1: PFS patterns of patients in Subgroup B (1st line, chemo-naïve + pre-treated) with mRNA RQ values classified in a 3-scale as low, normal and high. Figure 2: Significant but non-specific associations of BRCA1 and RRM1 gene expression with the outcome of 1st line chemo-naïve patients treated with taxanes excluding the platinum doublet. Figure 3: Effect of ERCC1 mRNA expression on the outcome of 1st line chemo-naïve treated patients. [file 1471-2407-12-342-S1.pdf]

Expression of DNA repair and replication genes in Non-Small Cell Lung Cancer (NSCLC): a role for Thymidylate Synthetase (TYMS)

Kotoula V, Krikelis D, *et al*

ADDITIONAL TABLES and FIGURES

Additional Table 1. Clinicopathologic characteristics of NSCLC patients and tumors examined.

**Available tumor blocks, N=321**		
**Available normal lung* blocks, N=40**		
**Age (years), N=321**		
median	63,8	
min	35,7	
max	80,0	
	**N**	**%**
**Gender**	**321**	
Men	266	81,6
Women	60	18,4
**Histology**	**321**	
Adenocarcinoma	173	53,1
SCC	76	23,3
LCC/undifferentiated	72	22,1
**mRNA data, cases**	**281**	
Matched T/N	35	12,5
Tumors only	241	85,8
Normal only	5	1,8
**All treated patients**	**241**	
**Smoking status**	**209**	
Never	30	14,4
Former	26	12,4
Current	153	73,2
**Surgery**	**227**	
Yes	93	41,0
No	134	59,0
**Stage at initial diagnosis**	**230**	
I & II	54	23,5
IIIA	34	14,8
IIIB & IV	142	61,7

*: distal to tumor; SCC and LCC: squamous and large cell carcinoma; matched T/N: matched tumor / normal lung tissue samples

Additional Table 2: Treatment-related patient groups and outcome.

			PFS / DFS* (months)		OS (months)	
	N	%	nr of events	median	nr of events	median
**Initial treatment, all patients**	**216**					
**Settings**						
adjuvant	46	21,3	26	14,9	8^	35,9
neoadjuvant	10	4,6	6	7,7	3^	9,3
Subgroup A	160	74,1	128	6,9	102	13,4
Subgroup B	*180*	*83,3*	*141*	*7,2*		
Subgroup C	*192*	*88,9*			*110*	*21,6*
**Regimens**						
platinum-containing, no taxane doublet						
Subgroup A	25/160	15,6	20	7,3	18	17,1
Subgroup B	27/180	15,0	21	7,3		
Subgroup C	28/192	14,6			19	18,0
taxane-containing, no platinum doublet						
Subgroup A	70/160	43,8	61	4,1	52	13,7
Subgroup B	76/180	42,2	66	4,2		
Subgroup C	71/192	37,5			53	13,5
taxane + platinum (TAXPLAT) regimen						
Subgroup A	51/160	31,9	37	10,4	25	20,9
Subgroup B	57/180	31,7	41	10,1		
Subgroup C	78/192	40,6			31	43,6

Subgroup A: 1st line, chemo-naïve; Subgroup B: 1st line, chemo-naïve and pre-treated; Subgroup C: chemo-naïve, 1st line and non-relapsed adjuvant; *: prograssion free survival (PFS) in the 1st line, disease free survival (DFS) in the adjuvant setting; OS: overall survival; ^: OS was calculated for patients who received no more than adjuvant (n = 32) or neoadjuvant treatment (n=4); SD = standard deviation

Additional Table 3: Selection of the normal RQ value range was based on the highest frequency of RQ values within a 3-cycle range in the normal sample series.

RQ range	BRCA1	ERCC1	RRM1	TYMS
<34	1	1	1	17
34 to <35	3		2	4
35 to <36	13		8	7
36 to <37	15	3	3	10
37 to <38	4	3	6	2
38 to <39	3	12	12	
39 to <40		17	4	
40 to <41	1	2	2	
41 to <42			2	
42 to <43		2		
total N	40	40	40	40

Additional Table 4: Initial classification of B, E, R, T RQ values in NSCLC tumors.

	**BRCA1**	**ERCC1**	**RRM1**	**TYMS**
RQ values normal range, N tumors	**184** (67.15%)	**193** (69.93%)	**162** (58.70%)	**139** (50.38%)
RQ values below normal range, N tumors, A	23	25	61	26
RQ values below normal minimum, N tumors	*2*	*6*	*3*	*0*
RQ values above normal range, N tumors, B	67	58	53	111
RQ values above normal maximum, N tumors	*7*	*1*	*14*	*90*
aberrant RQ values, N tumors, A + B	**90** (32.85%)	**83** (30.07%)	**114** (41.30%)	**137** (49.64%)
all RQ values	**274**	**276**	**276**	**276**

Additional Figure 1

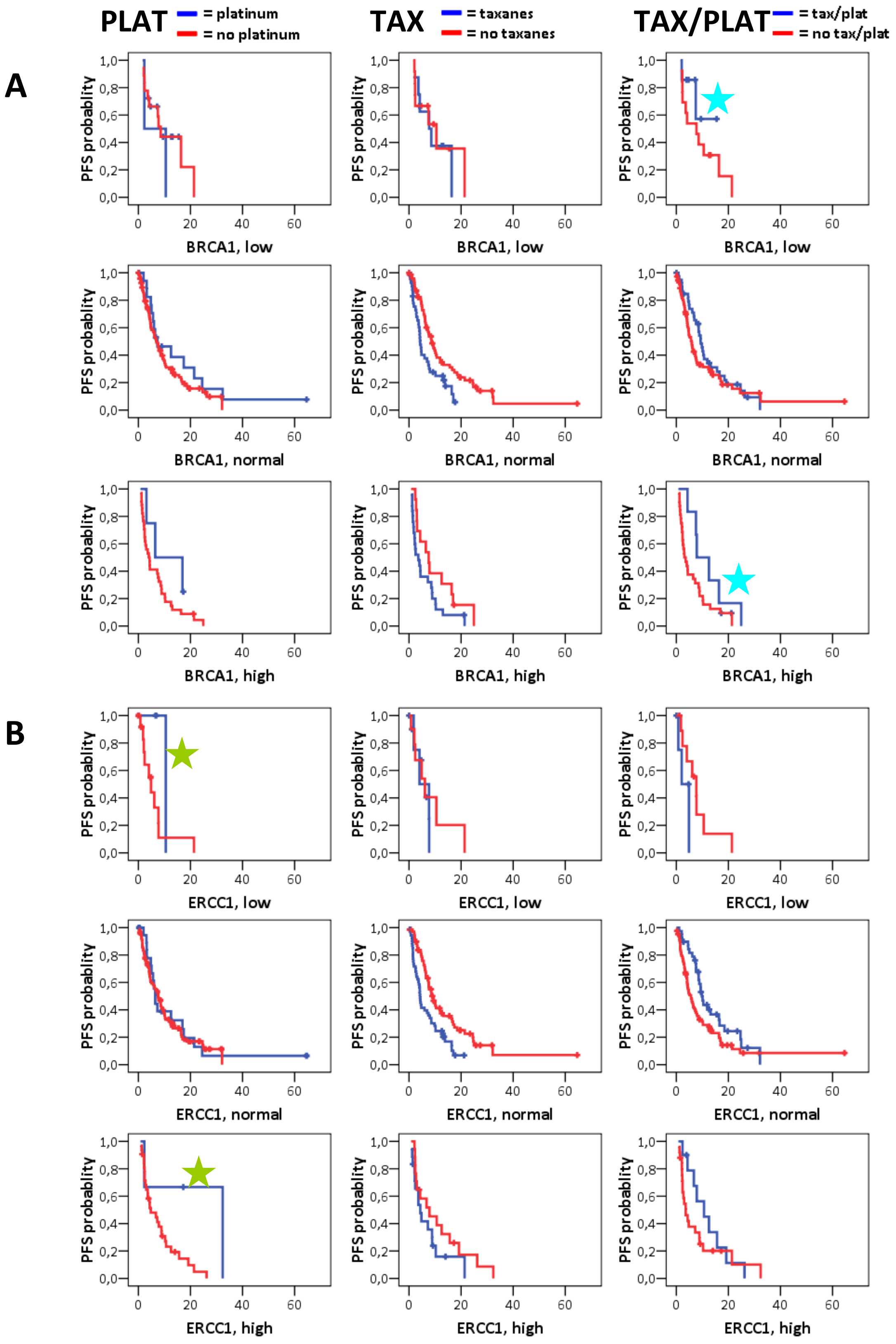

Additional Figure 1 (continued)

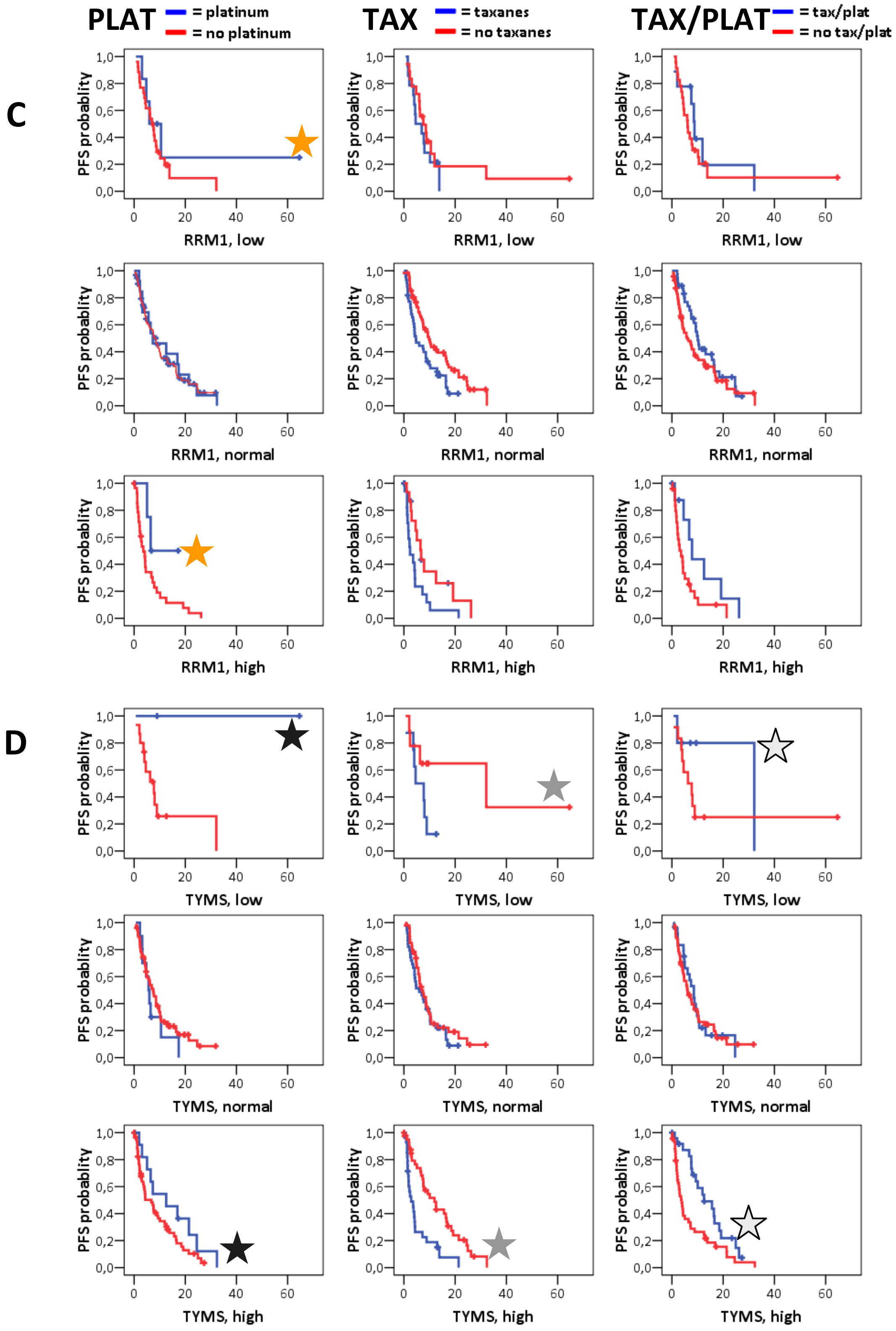

Additional Figure 1: PFS patterns of patients in Subgroup B (1st line, chemo-naïve + pre-treated) with mRNA RQ values classified in a 3-scale as low, normal and high. Low and high stands for RQ values lower and higher than normal, respectively, while normal RQ values are included in a range of 3 cycles. Kaplan-Meier curves for all markers (**A,** BRCA1; **B,** ERCC1, **C,** RRM1, **D,** TYMS) with patients grouped vertically according to the 3 main chemotherapy regimens: left panels, platinum without taxanes; middle panels, taxanes without platinum; right panels, taxanes/platinum doublet. X-axes: time in months. Paired color stars indicate similar PFS patterns for low and high RQ values per marker. The apparent better prognosis for normal BRCA1 and ERCC1, as well as for low RRM1 and, especially, low TYMS did not reach statistical significance.

Additional Figure 2

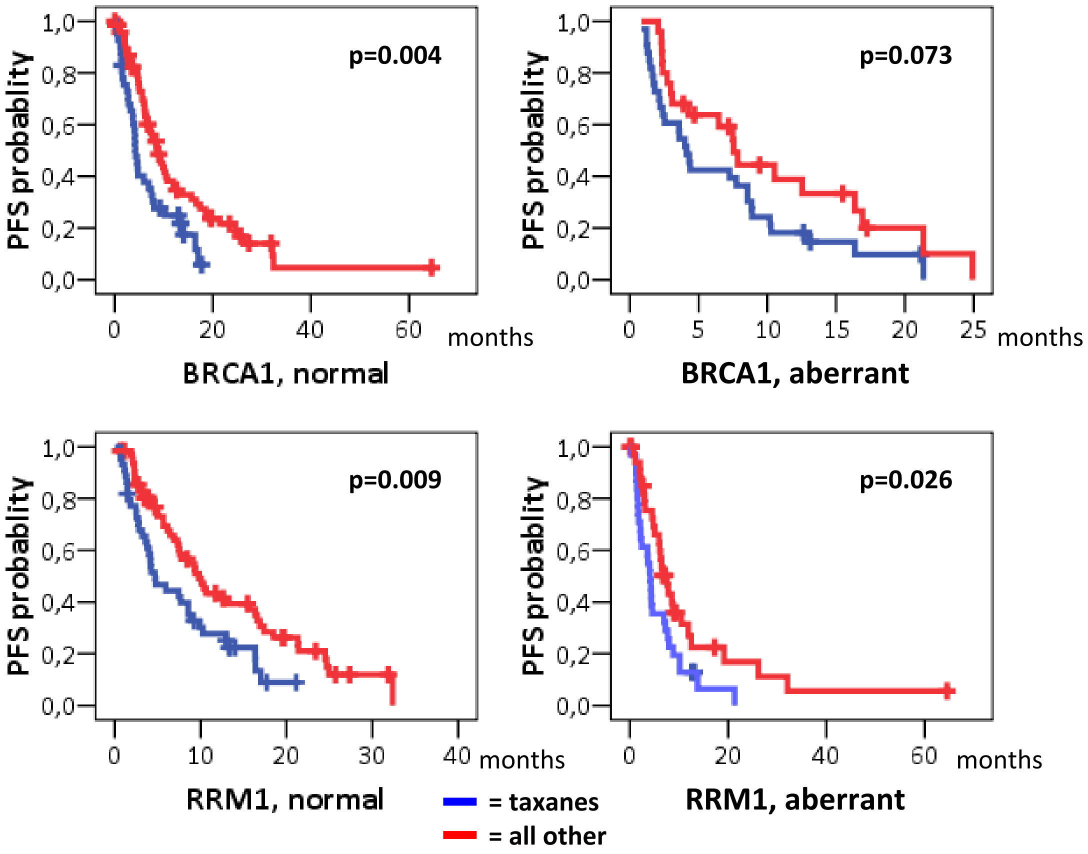

Additional Figure 2: Significant but non-specific associations of BRCA1 and RRM1 gene expression with the outcome of 1st line chemo-naïve patients treated with taxanes excluding the platinum doublet. Although BRCA1 and RRM1 may appear significant for the outcome upon taxane treatment, such univariate findings were not specific for this drug.

Additional Figure 3

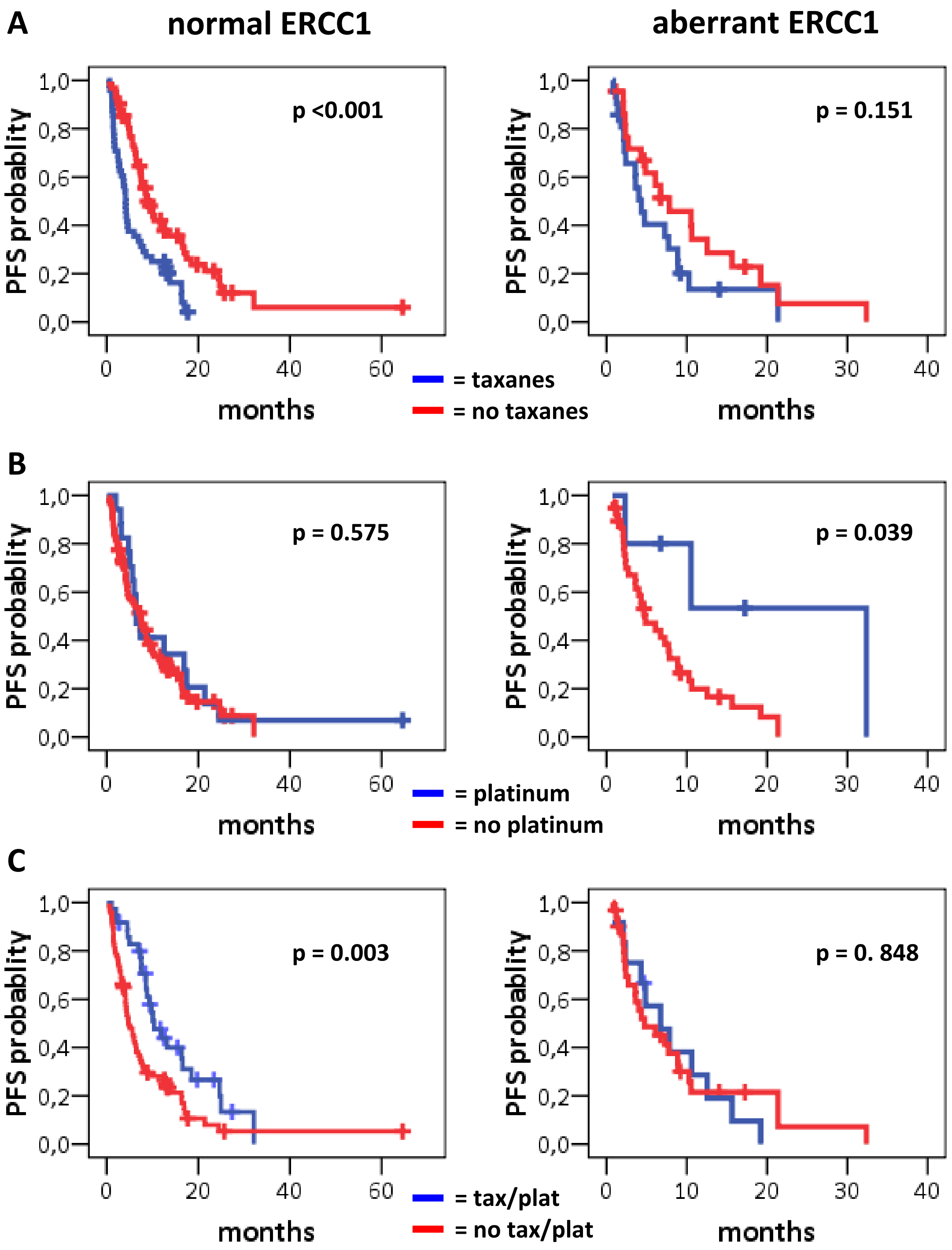

Additional Figure 3: Effect of ERCC1 mRNA expression on the outcome of 1st line chemo-naïve treated patients. Normal ERCC1 was unfavorable for taxanes excluding the taxanes/platinum combination (A), while aberrant ERCC1 was favorable for the 5 patients who received platinum regimens, again excluding the

tax/plat combination (B). With respect to the taxanes/platinum combination, normal ERCC1 was associated with a favorable PFS in patients who received this combination in comparison to those who did not. Log-rank p's are shown.
